# Progressive failure analysis of perforated composite laminates considering nonlinear shear effect

**DOI:** 10.1038/s41598-023-49823-6

**Published:** 2023-12-16

**Authors:** Z. R. Wu, Yirong Yang, Hang Lei

**Affiliations:** 1 State Key Laboratory of Mechanics and Control of Mechanical Structures, Nanjing University of Aeronautics and Astronautics, Nanjing, 210016 Jiangsu China; 2 College of Energy and Power Engineering, Nanjing University of Aeronautics and Astronautics, Nanjing, 210016 Jiangsu China; 3 AVIC Chengdu Aircraft Design and Research Institute, 1610 Riyue Avenue, Qingyang District, Chengdu, 610091 Sichuan China

**Keywords:** Aerospace engineering, Composites

## Abstract

Composites are widely used in high performance structures such as aerospace structures due to their excellent properties. The analysis of failure evolution of composite perforated structures by finite element simulation is of great significance for practical work as engineering composite structures often contain notches and voids. In this paper, the numerical simulation of failure evolution and failure modes of carbon fiber reinforced resin composite laminates with large openings was carried out. A UMAT subroutine was written based on the 3D Hashin-Ye failure criterion and progressive damage model theory. The characteristic length and viscosity coefficient were introduced into the model to reduce mesh dependency and improve computational convergence. The nonlinear shear constitutive relationship defined by the Ramberg–Osgood equation was introduced into the continuous damage degradation model. The effect of nonlinear shear on the failure evolution of laminates with different stacking sequence was studied.

## Introduction

There are many types of composite structures applied in practical engineering that can be considered as composite laminates with large openings. The relatively large size of the opening seriously damages the continuity of the fibers and exacerbates the stress concentration in the composite laminates. In order to ensure the safety of composite structures, it is necessary to explore the failure mechanism of large opening laminates through failure analysis and damage evolution.

**Figure 1:**
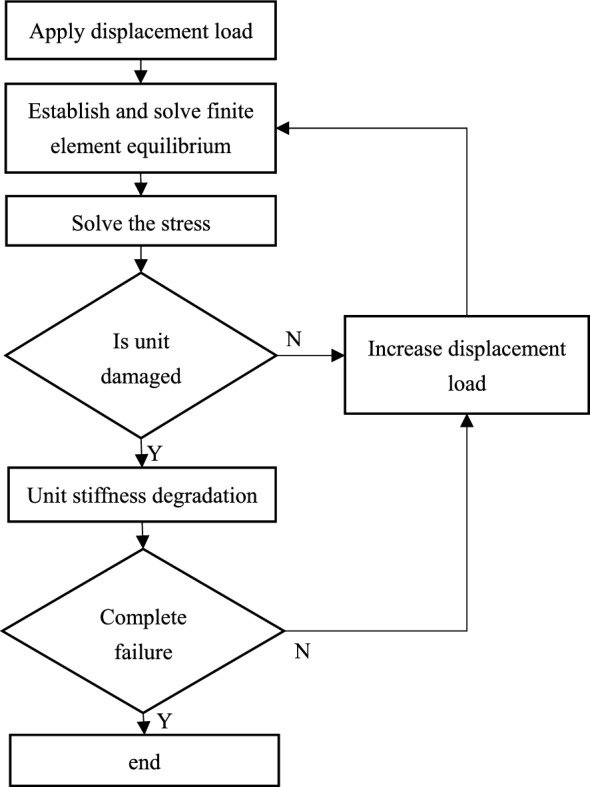
Progressive failure analysis flow chart.

There are three main failure analysis methods for composite structures. The damage analysis methods are based on fracture mechanics, damage area model and progressive damage model respectively. The failure analysis method for composite structures using progressive damage model is a numerical failure theory. The progressive damage analysis method is a calculation method based on the assumption that the damaged material can continue to be loaded according to its properties after degradation. Shabani proved that by having unidirectional properties of a composite, a progressive fatigue damage model was capable of estimating the static strength of the notched rings with less than 5% deviation^1^. The Ochoa study fully shows the basic steps of progressive failure of composites as shown in Fig. 1. This method is usually divided into four steps: (1) Analyze the strains and stresses in single-ply plates. The problem is the establishment of constitutive equation, which is usually calculated by means of finite element method. (2) The failure criterion of composite material is used to judge whether the single-layer plate has failed or not. If not, then continue to increase the load to repeat the above calculation, and if there is a failure, proceed to the next step. (3) Progressive failure analysis after materials have failed. Attenuation of the material stiffness of a failed single-layer plate is usually performed by the laminar reduction method and by the continuous damage mechanics, which are described in detail below. (4) To judge the total failure of the laminated plate structure.

Many scholars have proposed a large number of failure criteria in the past few decades. The representative ones are Tsai-Wu criterion^2^, Hashin criterion^3^, Puck criterion^4–6^, etc. Failure criteria can be roughly classified into two categories, one is the criterion that does not differentiate between different failure modes and the other that is associated with failure modes. The most commonly used polynomial failure criterion is proposed by Tsai and Wu^2^. Other outstanding secondary failure criteria include Hill^7^, Tsai Hill^8^, Hoffman^9^ and Chamis^10^. Criteria depending on failure mode include Hart Smith^11^, Hashin–Rotem^3^, Christensen^12^, puck^4–6^, etc.

Hashin proposed Hashin failure criterion in 1980, which was based on the original Hashin–Rotem criterion. The classification of failure modes had been increased from 2 to 4, which are fiber tensile failure, fiber compression failure, matrix tensile failure and matrix compression failure. This criterion provided a theoretical basis for subsequent failure criteria related to different failure modes. Hahin criterion has been applied in many commercial finite element analysis software and is still a widely used failure criterion up to now.

The damage can be considered as allowable damage in certain failure modes when only one layer of the overall composite structure is damaged or partially damaged. And the structure can still withstand loads without causing the whole structure to fail. The load will be redistributed between adjacent layers. The overall properties (strength, stiffness, etc.) of the composites will gradually decrease with the accumulation of damage from a macro perspective. Therefore, it is necessary to construct a degradation model of composite properties when failure analysis of composite laminates is carried out. According to the degradation mode of damaged composites, stiffness degradation models of composites can be divided into two categories^13^: ply-discounting degradation model and continuum damage model (CDM).

The ply-discounting degradation model is a model of sudden rigidity degradation. The material properties of a particular unit are reduced to a certain value when damage occurs, but the accumulation and expansion of damage are not taken into account. The Continuum Damage Model (CDM) is a model of continuous stiffness degradation. The coefficient in the constitutive relationship is set as a function of one or more internal state variables by defining one or more built-in state variables to describe the internal damage of a material in CDM. Damage begins to occur in the composite material, which leads to a decrease in the overall stiffness of the material. The stiffness of the material decreases continuously as damage accumulates. The Continuous Damage Model effectively avoids the uncertainty of material degradation coefficient in the ply-discounting degradation model. All parameters applied in this model have clear physical meaning and can be measured directly. And it has been proven to be an effective and reliable method for simulating laminate damage.

Although fibrous composite materials are basically designed to make use of the stiffness and strength of the fibers, the matrix is also subjected to different loading conditions. Therefore, they exhibit significant nonlinear mechanical behavior in the matrix-dominated loading conditions due to their polymeric matrix^13^. An extensive numerical study had been conducted in order to investigate the effects of material nonlinearity on the stress distribution and stress concentration factors in unidirectional and laminated composite materials^14^. A progressive fatigue damage model is proposed by considering the nonlinearity effects of in-plane shear stress/strain relationship^15^. Fallahi presented a broad perspective on the main factors controlling the material nonlinearity within a simple constitutive model^16^.

In this paper, a numerical simulation method for tensile failure of composite laminates considering nonlinear shear has been established. The simulation accuracy of the layer reduction material degradation model and the continuous degradation model was compared. The influence of considering nonlinear shear on the simulation accuracy of laminates with different layers and different sizes of openings was studied. Finally, the test results are compared with the simulation results to verify the rationality of the simulation.

## Compilation of user-material subroutine

### Compilation principle

The user defined material subroutine (UMAT) is used to define the mechanical behavior of a new material^17^. It is a very flexible, practical, and convenient method suitable for damage calculation of composite materials. Its main characteristics include: (1) It can be used to define the constitutive model of new materials. (2) It is invoked at all material points of the unit of user-defined material for each incremental step. (3) Solution dependent state variables can be used. (4) The stress and state variables can be updated at the end of the incremental step. (5) A Jocobian matrix of material constitutive relations can be provided.

1 $$\frac{\partial \sigma }{{\partial \varepsilon }} = C_{d} + \frac{{\partial C_{d} }}{\partial \varepsilon }:\varepsilon$$

The expression of the damage stiffness matrix *C*_*d*_ is

2 $$\left[ {C_{d} } \right] = \left[ {\begin{array}{*{20}l} {C_{11}^{d} } & {C_{12}^{d} } & {C_{13}^{d} } & 0 & 0 & 0 \\ {C_{21}^{d} } & {C_{22}^{d} } & {C_{23}^{d} } & 0 & 0 & 0 \\ {C_{31}^{d} } & {C_{32}^{d} } & {C_{33}^{d} } & 0 & 0 & 0 \\ 0 & 0 & 0 & {C_{44}^{d} } & 0 & 0 \\ 0 & 0 & 0 & 0 & {C_{55}^{d} } & 0 \\ 0 & 0 & 0 & 0 & 0 & {C_{66}^{d} } \\ \end{array} } \right]$$

Substituting it into Formula (1) can obtain:

3 $$\frac{\partial \sigma }{{\partial \varepsilon }} = C_{d} + \frac{{\partial C_{d} }}{{\partial d_{i} }}\frac{{\partial d_{i} }}{\partial \varepsilon }:\varepsilon \left( {i = 1,2,3} \right)$$

**Figure 2:**
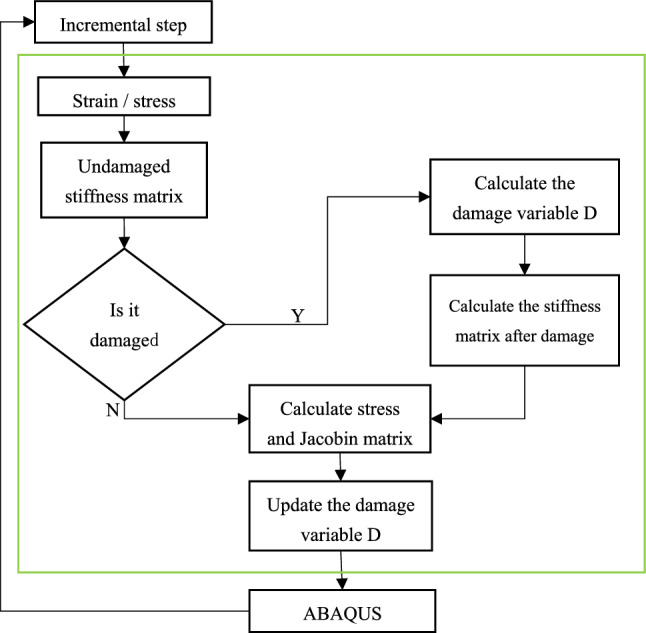
UMAT calculation flow chart.

The user material subroutine (UMAT) flowchart is shown in Fig. 2. A set of equations was established for the entire model and then solved to obtain a displacement convergent solution assuming that the material state unchanged in each load increment step. Substitute the calculated stress/strain status of each material point into the undamaged stiffness matrix to obtain the updated stress/strain. The updated stress/strain is then substituted into the failure criterion to determine whether damage has occurred. If no damage occurs, continue to increase the displacement load after calculating the stress and proceed to the next incremental step. If any damage failure occurs, calculate the damage variable *d* and the Jocobian matrix after the damage, and finally return to ABAQUS. The same process is performed at each incremental step until the material has completely failed.

### Characteristic length method for eliminating mesh dependency

The material will exhibit strain softening behavior in the calculation, leading to strain localization. This can lead to strong mesh dependency in the finite element results. Characteristic length along with failure criteria were used to predict strength of the notched composite plates subjected to the in-plane tensile loading. Lapczyk^18^ has demonstrated the phenomenon that the dissipation energy is proportional to the volume of the failed element through an example of the uniaxial tension of the rod. He used the crack zone model proposed by Bažant et al. in order to avoid situations where different element sizes yield different dissipation energies in the results^19^. The fracture was simulated as a parallel distributed tightly microcrack band in this model. The characteristic length of the element is introduced to regularize the calculated dissipation energy in order to eliminate the dependency of meshes, namely:

4 $$g_{i} = \frac{{G_{i} }}{Lc}\left( {i = 1,2,3} \right)$$

In the formula, *G*_*i*_ is the fracture energy, *L*_*c*_ is the unit characteristic length, and *g*_*i*_ is the fracture energy within the unit volume.

### Viscosity coefficient for improving convergence

It is very likely to encounter convergence difficulties due to the softening of materials and stiffness degradation behavior in calculation. The viscous regularization scheme proposed by Duvaut and Lions^20^ was used for damage variables to solve this problem. This method can make the tangent stiffness matrix of the softened material positive definite in a sufficiently small increment. The damage variable was regularized by the following equation:

5 $$\mathop {d_{i}^{\upsilon } }\limits^{ \bullet } = \frac{1}{\eta }\left( {d_{i} - d_{i}^{v} } \right)\left( {i = 1,2,3} \right)$$

In the formula, *d*_*i*_ represents the damage variables, and $d_{i}^{\nu }$ represents the regularized damage variables. The regularized damage variables are used to calculate the damage stiffness matrix and the Jacobian matrix in practical calculations. *η* is the viscosity coefficient, which represents the relaxation time in the viscous system. It controls the rate at which the regularized damage variable $d_{i}^{\nu }$ approaches the damage variable *d*_*i*_. The convergence rate of the model in the softening process can be improved when the ratio of viscosity coefficient to characteristic time increment is small. When ${t \mathord{\left/ {\vphantom {t {\eta \to \infty }}} \right. \kern-0pt} {\eta \to \infty }}$ (*t* represents time), the solution of a viscous system tends to be that of a non-viscous system. To calculate the regularized damage variables at time $t + \vartriangle t$, discretize Eq. (5) in time to obtain the following equation

6 $$d_{i}^{v} \left| {_{t + \vartriangle t} } \right. = \frac{\vartriangle t}{{\eta + \vartriangle t}}d_{i}^{{}} \left| {_{t + \vartriangle t} } \right.{ + }\frac{\eta }{\eta + \vartriangle t}d_{i}^{v} \left| {_{t} } \right.\left( {i = 1,2,3} \right)$$

It can be further deduced that

7 $$\frac{{\partial d_{i}^{v} }}{{\partial d_{i} }} = \frac{\vartriangle t}{{\eta + \vartriangle t}}$$

The final Jacobian matrix can be obtained as follows by substituting Eq. (7) into Eq. (6).

8 $$\frac{\partial \sigma }{{\partial \varepsilon }} = C_{d} + \left\{ {\sum\limits_{i = 1}^{3} {\left[ {\left( {\frac{{\partial C_{d} }}{{\partial d_{i}^{v} }}\frac{{\partial d_{i} }}{\partial \varepsilon }:\varepsilon } \right)\left( {\frac{{\partial d_{i} }}{{\partial f_{i} }}\frac{{\partial f_{i} }}{\partial \varepsilon }} \right)} \right]} } \right\}\frac{\vartriangle t}{{\eta + \vartriangle t}}\left( {i = 1,2,3} \right)$$

**Figure 3:**
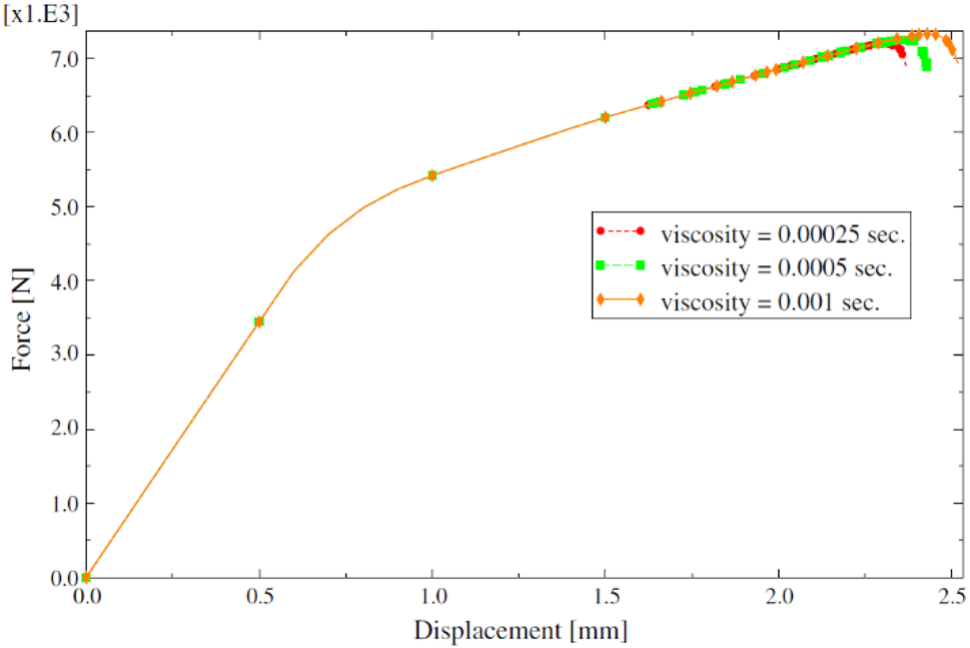
Force–displacement curves under different viscosity coefficients.

It is necessary to select an appropriate value of the viscosity coefficient *η* to ensure the accuracy of the results. Excessive viscosity coefficient can cause delays in material degradation. The research of Lapczyk^1^ shows the influence of different viscosity coefficients on the strength calculation results of laminates. It can be seen from Fig. 3 that the values of three different viscosity coefficients do not have a significant impact on the results, but they significantly improve the convergence of the calculation.

## UMAT numerical simulation of progressive damage degradation model

### Degradation model of material performance

Simulation methods can be established using ply-discounting degradation models when empirical curves or test data are available. It can simplify the calculation model, improve model convergence, and reduce calculation time. On the other hand, the continuum damage degradation model is based on the continuum damage mechanics theory, which can effectively avoid the uncertainty of the degradation coefficient in the ply-discounting degradation model. In this chapter, two degradation models will be used to simulate the failure process of composite laminates, and the ultimate load and load–displacement curves obtained will be compared with experimental data ^21^. The Chang–Chang model was selected as the degradation model for simulation based on the 3D Hashin criterion. And the exponential degradation was selected as the degradation mode for progressive failure. The performance degradation parameters of the Chang–Chang model are shown in Table 1.

**Table 1:** The performance degradation parameters of the Chang–Chang model.

Failure mode	Chang–Chang model
Tensile failure of fibers	$E_{11} = 0$
Compression failure of fibers	$E_{11} = 0$
Tensile failure of matrix	$E_{22} = G_{12} { = }G_{23} = 0$
Compression failure of matrix	$E_{22} = G_{12} { = }G_{23} = 0$

### Finite element simulation model

**Figure 4:**
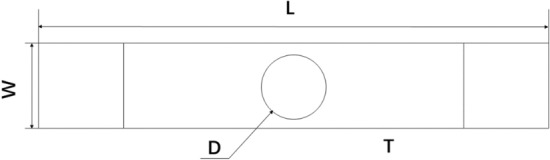
Geometric dimensions of the finite element model.

The geometric model was established as shown in Fig. 4, where W is the width of the laminate, L is the length of the laminate, D is the diameter of the central hole in the laminate, and T is the thickness of the laminate.

The geometric dimension parameters and stacking sequence are shown in Table 2. The composite laminates were made of T300/1034-C prepreg. The elastic constants and strength parameters of the material are shown in Tables 3 and 4.

**Table 2:** T300/1034-C composite laminate specimen geometric dimensions and stacking sequence^21^.

Stacking sequence	L (mm)	W (mm)	D (mm)	T (mm)
$\left[ {{0/}\left( { \pm {45}} \right)_{{3}} {/90}_{{3}} } \right]_{S}$	203.2	25.4	6.35	2.616

**Table 3:** Elastic coefficients of T300/1034-C composite material.

E_11_ (GPa)	E_22_ (GPa)	G_12_ (GPa)	E_F1_ (GPa)	v_12_	v_23_	V_F12_
146.9	11.4	6.18	230	0.3	0.4	0.2

**Table 4:** Strength parameters of T300/1034-C composite material.

X_T_ (MPa)	X_C_ (MPa)	Y_T_ (MPa)	Y_C_ (MPa)	S_12_ (MPa)	S_23_ (MPa)
1370.6	1379	66.5	268.2	133.8	100

A boundary condition was applied to the model with one end fixed and the other end coupled with a reference point. A tensile load was applied to the reference point, and the load displacement curve of the reference point was output. The displacement load of 2 mm was applied to the model.

### Comparison of two degradation criteria

**Figure 5:**
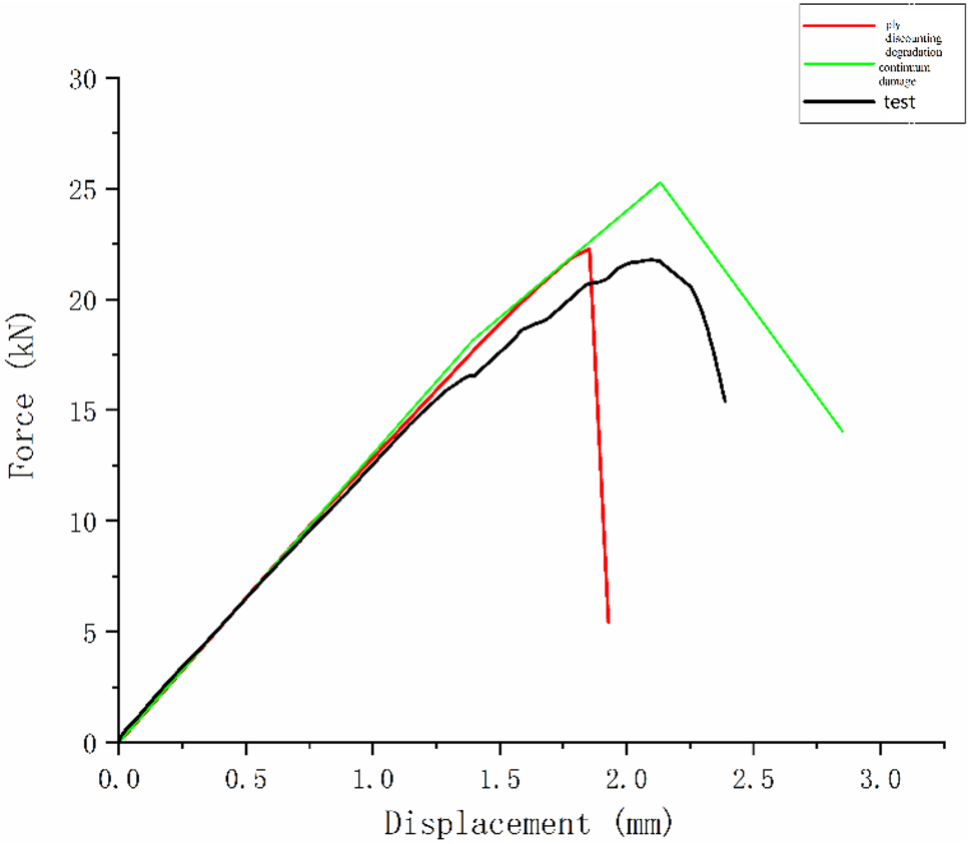
Load displacement curves of simulation using different damage models and the tests.

Figure 5 shows the load displacement curve of a laminate using the ply-discounting degradation model and the continuum damage model, respectively. Table 5 shows the error analysis between the calculated tensile failure strength of laminates and the test values in literature ^21^. The error of both sets of data is below 17%, which proves that the UMAT subroutine can meet the calculation requirements. The error of the ply-discounting degradation model is 16.98%, while the error of the continuum damage model is only 3.05%. It can be found that the load displacement curve obtained using the continuum damage model is closer to the test load displacement curve than using the ply-discounting degradation model comparing the results of the reduced damage model with the continuum damage model. Therefore, the continuum damage model will be used in the following simulation research.

**Table 5:** The error of T300/1034-C laminates’ ultimate loads.

Type of model	Value of simulation (kN)	Value of tests (kN)	Error (%)
Ply-discounting degradation model	25.28	21.61	16.98
Continuum damage model	22.27	21.61	3.05

## Simulation and verification of perforated laminates

### Finite element model of perforated laminates

**Figure 6:**
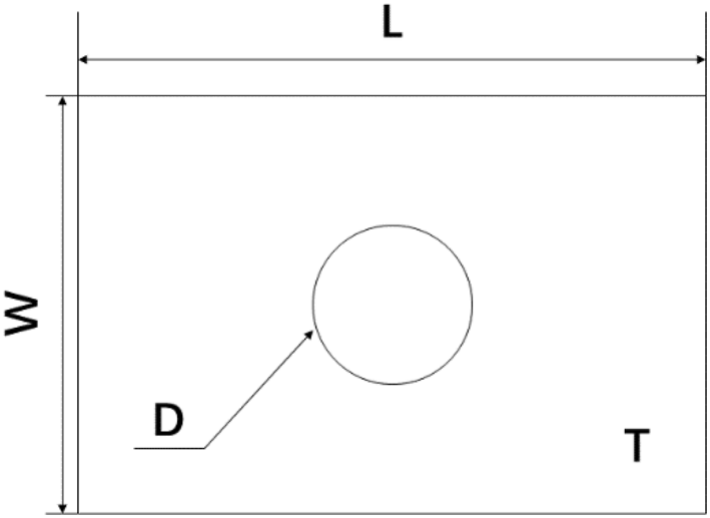
The geometric model of the perforated laminates.

The geometric model of the perforated laminates is shown in Fig. 6. The geometric dimensions and fiber stacking sequence of three different plies and three different pore diameters of perforated composite laminates are shown in Table 6. The elastic constants and strength parameters of materials are shown in Tables 7 and 8.

**Table 6:** The geometric dimensions and fiber stacking sequence of perforated laminates.

No.	Stacking sequence	L (mm)	W (mm)	D (mm)	T (mm)
A-1	$\left[ {0/90} \right]_{5}$	240	160	100	1.58
A-2	$\left[ {0/90} \right]_{5}$	240	160	80	1.58
A-3	$\left[ {0/90} \right]_{5}$	240	160	60	1.58
B-1	$\left[ {0} \right]_{10}$	240	160	100	1.58
B-2	$\left[ {0} \right]_{10}$	240	160	80	1.58
B-3	$\left[ {0} \right]_{10}$	240	160	60	1.58
C-1	$\left[ { \pm {45}} \right]_{5}$	240	160	100	1.58
C-2	$\left[ { \pm {45}} \right]_{5}$	240	160	80	1.58
C-3	$\left[ { \pm {45}} \right]_{5}$	240	160	60	1.58

**Table 7:** The elastic constants of the composite material.

E_11_ (GPa)	E_22_ (GPa)	G_12_ (GPa)	v_12_	v_23_
127.7	9.67	6.5	0.3	0.4

**Table 8:** The strength parameters of the composite material.

X_T_ (MPa)	X_C_ (MPa)	Y_T_ (MPa)	Y_C_ (MPa)	S_12_ (MPa)	S_23_ (MPa)
1528.7	764.3	36.0	160.3	98	133

The boundary conditions and displacement load applied to the finite element model in this section were the same as in the previous chapter.

### Failure evolution and failure mode analysis

**Figure 7:**
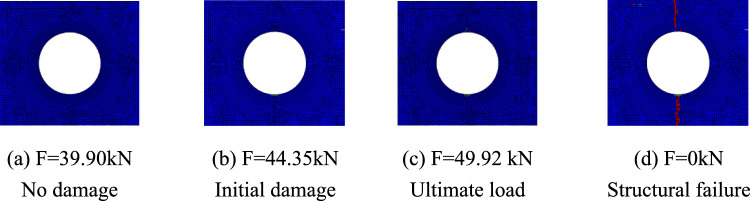
The damage propagation of fiber failure of the 0° layer in the $\left[ {0/90} \right]_{5}$ laminate (D = 80 mm).

**Figure 8:**
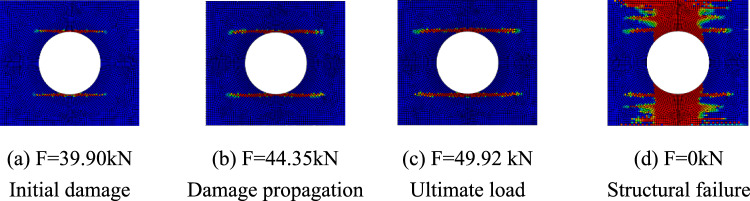
The damage propagation of matrix failure of the 0° layer in the $\left[ {0/90} \right]_{5}$ laminate (D = 80 mm).

**Figure 9:**
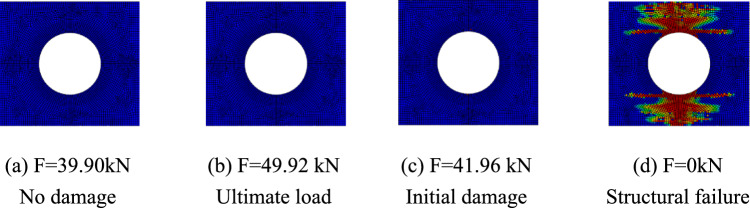
The damage propagation of delamination of the 0° layer in the $\left[ {0/90} \right]_{5}$ laminate (D = 80 mm).

**Figure 10:**
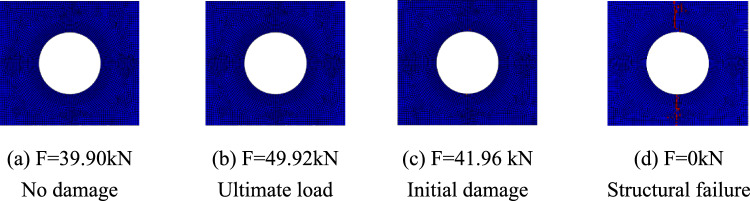
The damage propagation of fiber failure of the 90° layer in the $\left[ {0/90} \right]_{5}$ laminate (D = 80 mm).

**Figure 11:**
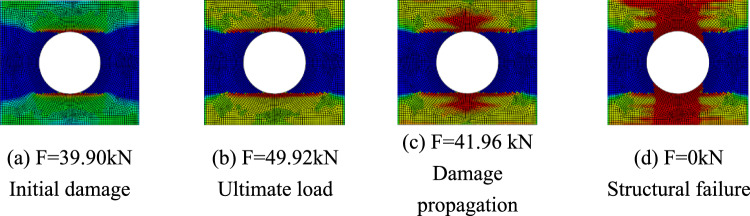
The damage propagation of matrix failure of the 90° layer in the $\left[ {0/90} \right]_{5}$ laminate (D = 80 mm).

**Figure 12:**
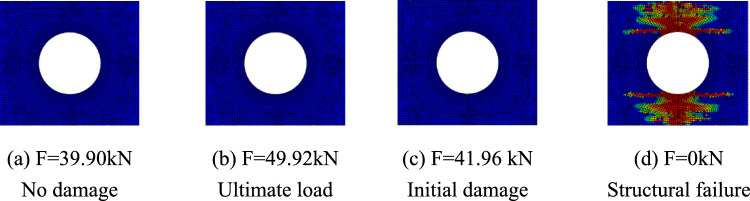
The damage propagation of delamination of the 90° layer in the $\left[ {0/90} \right]_{5}$ laminate (D = 80 mm).

The progressive failure process of composite laminates with large openings was analyzed taking the simulation results of simulation models A-2, B-2, and C-2 as examples. The red units represent units that have completely failed, the blue units represent units that have not been damaged, and the darker the color of the unit, the more severe the damage. Figure 7 shows the damage propagation of fiber failure of the 0° layer in the $\left[ {0/90} \right]_{5}$ laminate, Fig. 8 shows that of matrix failure and Fig. 9 shows the delamination failure. Figure 10 shows the damage propagation of fiber failure of the 90° layer in the $\left[ {0/90} \right]_{5}$ laminate, Fig. 11 shows the matrix failure and Fig. 12 shows the delamination failure.

**Figure 13:**
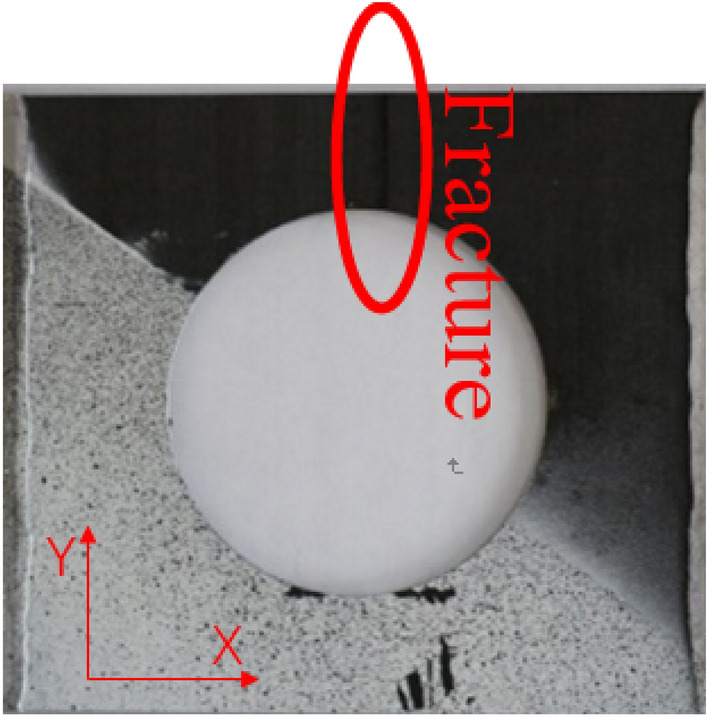
The tensile test failure of $\left[ {0/90} \right]_{5}$ laminate (D = 80 mm)^17^.

**Figure 14:**
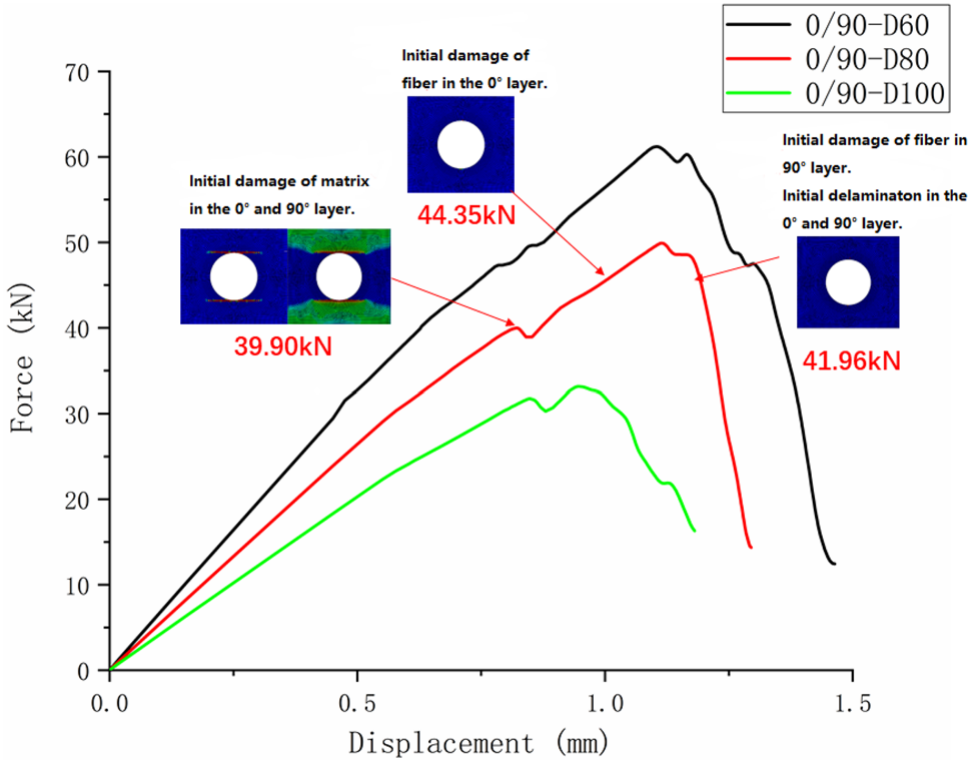
The load–displacement curves of $\left[ {0/90} \right]_{5}$ orthogonal laminates.

Figure 13 shows the tensile test failure of an 80 mm aperture orthogonal ply composite laminate^22^. Figure 14 shows the load–displacement curves of three groups of $\left[ {0/90} \right]_{5}$ orthogonal laminates with different damage apertures.

It can be found from the above figures that the 0° layer and 90° layer of the laminate first exhibited matrix damage (39.9 kN), followed by fiber damage (44.35 kN) in the 0° layer. Fiber and delamination failures (41.96 kN) occurred in the 90° layer in the process of significant structural performance failure, and delamination damage in the 0° layer also occurred with structural failure. The matrix damage first propagated along the loading direction, and then propagated in a direction perpendicular to the loading direction until it reached the edge of the structure. The fiber and delamination damage propagated in the direction perpendicular to the loading direction to the edge of the structure.

It can be found that the crack initiation positions of orthogonal laminated composite plates in simulation and experiment were all stress concentration points around the hole, and the failure mode was also a transverse fracture failure perpendicular to the tensile direction. Table 9 shows the error between simulation and test of three groups of orthogonal ply composite laminates with different damage apertures. It can be found that the three sets of simulations can accurately fit the tests, with the errors within 12%.

**Table 9:** Error in tensile strength of $\left[ {0/90} \right]_{5}$ composite laminates obtained through simulation and test ^22^.

No.	Stacking sequence	D (mm)	Strength by simulation (kN)	Strength by test (kN)	Error (%)
A-1	$\left[ {0/90} \right]_{5}$	100	33.15	37.54	− 11.69
A-2	$\left[ {0/90} \right]_{5}$	80	49.92	47.20	5.76
A-3	$\left[ {0/90} \right]_{5}$	60	61.20	59.32	3.17

**Figure 15:**
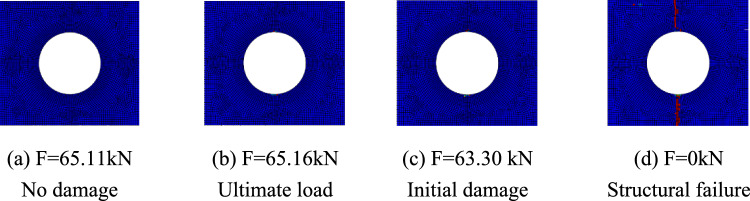
The damage propagation of fiber failure of the $\left[ {0} \right]_{10}$ laminate (D = 80 mm).

**Figure 16:**
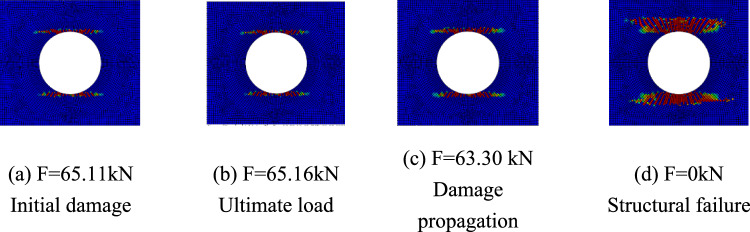
The damage propagation of matrix failure of the $\left[ {0} \right]_{10}$ laminate (D = 80 mm).

**Figure 17:**
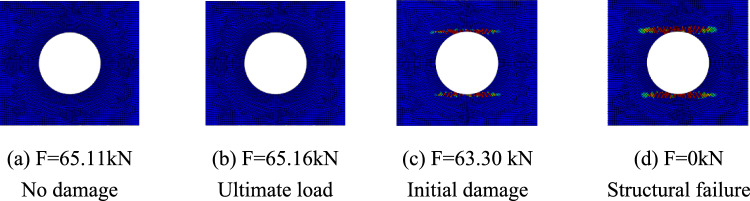
The damage propagation of delamination of the $\left[ {0} \right]_{10}$ laminate (D = 80 mm).

**Figure 18:**
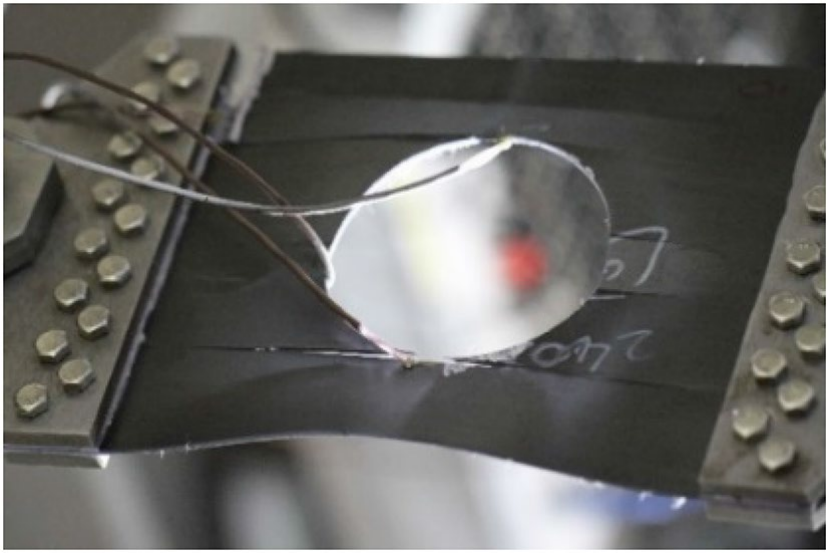
Failure diagram for tensile test of D = 80mm $\left[ {0} \right]_{10}$ laminate ^17^.

**Figure 19:**
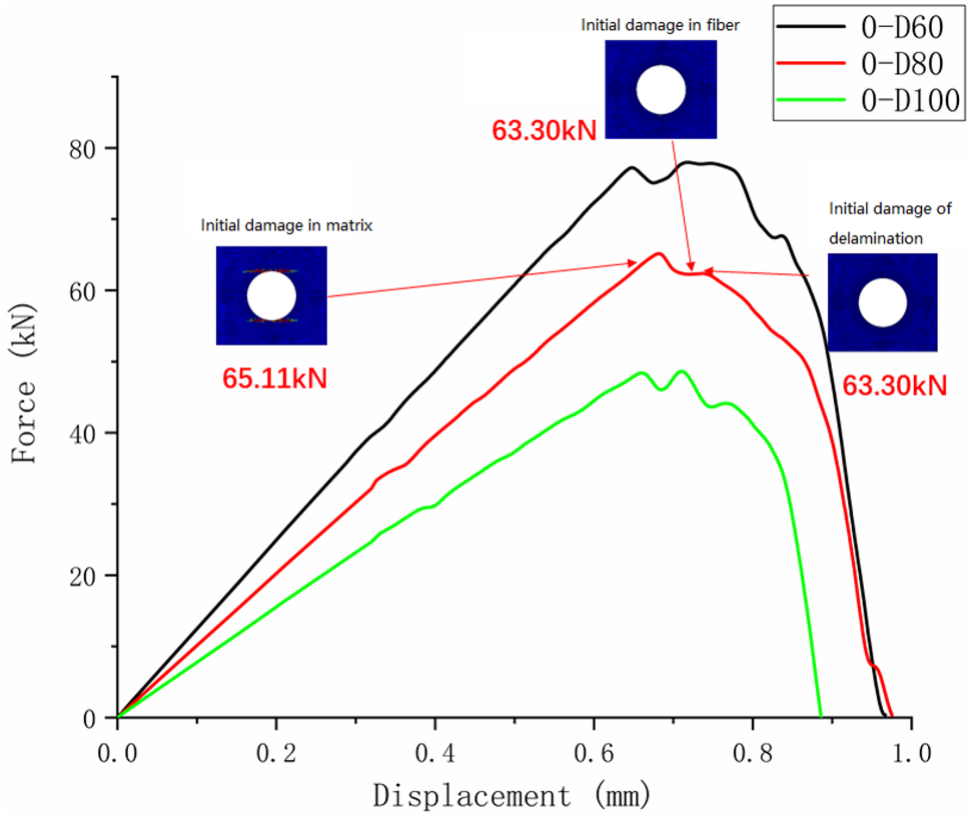
Load displacement curve of the $\left[ {0} \right]_{10}$ laminate.

Figures 15, 16, and 17 show the fiber failure propagation, matrix failure propagation and delamination failure propagation of $\left[ 0 \right]_{10}$ composite laminates, respectively. Figure 18 shows the failure diagram of the $\left[ 0 \right]_{10}$ unidirectional laminates with an 80 mm aperture during tensile testing ^22^. Figure 19 shows the load displacement curves of three groups of unidirectional laminates with different damage apertures.

It can be found that the matrix tensile damage (65.11 kN) first occurred, the failure location was at the hole edge, and the matrix damage expanded along the tensile direction. Fiber damage and delamination damage begin to occur (63.30 kN) as the load increases. The crack initiation position in both simulation and test was the stress concentration point at the hole edge. The failure mode was tensile damage to the matrix parallel to the tensile direction, resulting in relative slip and delamination failure. Table 10 shows the errors between simulation and test of three groups of $\left[ 0 \right]_{10}$ composite laminates with different damage apertures. It can be found that only B-1 has a large error and cannot be accurately fitted. The reason may be that the buckling phenomenon caused by excessive aperture was not considered. The relative errors of B-2 and B-3 were both within 7%.

**Table 10:** Error in tensile strength of $\left[ 0 \right]_{10}$ composite laminates obtained through simulation and test ^22^.

No.	Stacking sequence	D (mm)	Strength by simulation (kN)	Strength by test (kN)	Error (%)
B-1	$\left[ {0} \right]_{10}$	100	48.64	35.00	38.97
B-2	$\left[ {0} \right]_{10}$	80	65.16	69.73	− 6.55
B-3	$\left[ {0} \right]_{10}$	60	77.97	78.23	− 0.33

**Figure 20:**
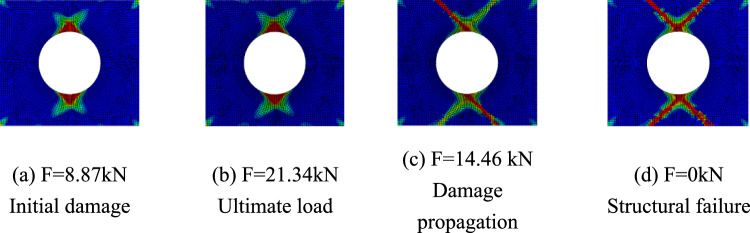
The matrix failure propagation of the 45° ply in the $\left[ { \pm 45} \right]_{5}$ laminate (D = 80 mm).

**Figure 21:**
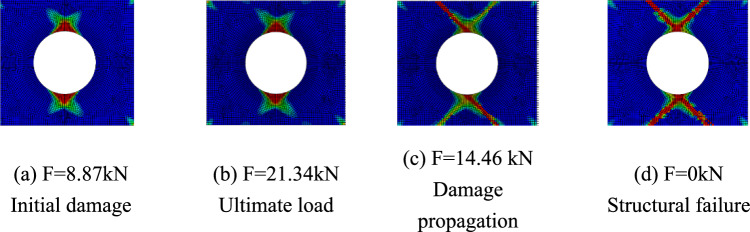
The matrix failure propagation of the -45° ply in the $\left[ { \pm 45} \right]_{5}$ laminate (D = 80mm).

**Figure 22:**
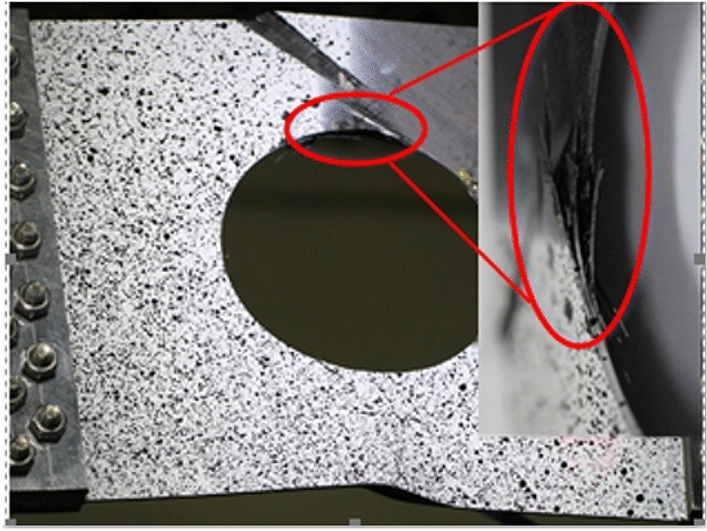
Failure diagram for tensile test of D = 80mm $\left[ { \pm {45}} \right]_{5}$ laminate^22^.

**Figure 23:**
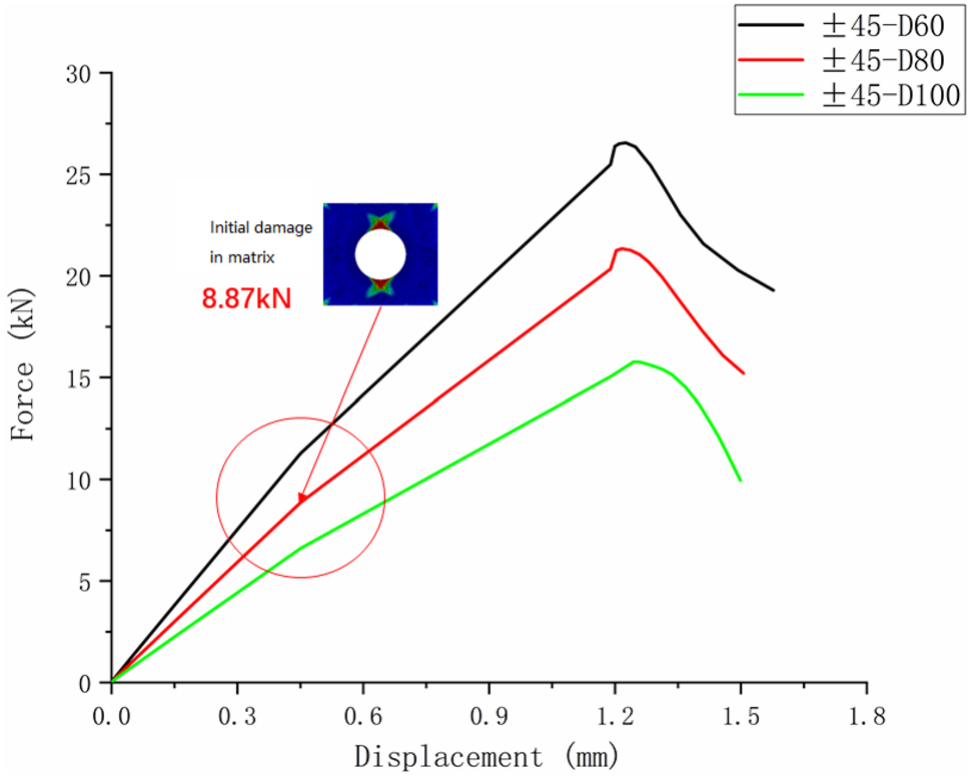
Load displacement curve of the $\left[ { \pm 45} \right]_{5}$ laminate.

Only matrix failure occurred in the $\left[ { \pm 45} \right]_{5}$ laminate. Figure 20 shows the matrix failure propagation of the 45° ply in the $\left[ { \pm 45} \right]_{5}$ laminate, and Fig. 21 shows the matrix failure propagation of the—45° ply in the laminate. Figure 22 shows the failure of the $\left[ { \pm 45} \right]_{5}$ orthogonal ply composite laminate with an aperture of 80 mm in tensile test ^22^. Figure 23 shows the load displacement curves of three groups of $\left[ { \pm 45} \right]_{5}$ laminates with different damage apertures.

It can be found that matrix damage occurred at the hole edge as the load increased(8.87 kN). The matrix damage propagated along the 45° direction from the tensile direction to the edge of the structure. The crack initiation position and failure mode of both simulation and test were the same.

Table 11 shows the errors between simulation and test of three groups of $\left[ { \pm 45} \right]_{5}$ composite laminates with different damage apertures. It can be found that the three sets of simulations can accurately fit the test, with the errors within 11%.

**Table 11:** Error in tensile strength of $\left[ { \pm 45} \right]_{5}$ composite laminates obtained through simulation and test^22^.

No.	Stacking sequence	D (mm)	Strength by simulation (kN)	Strength by test (kN)	Error (%)
C-1	$\left[ { \pm {45}} \right]_{5}$	100	15.77	17.60	− 10.40
C-2	$\left[ { \pm {45}} \right]_{5}$	80	21.34	23.79	− 10.30
C-3	$\left[ { \pm {45}} \right]_{5}$	60	26.55	28.72	− 7.56

Comparing Figs. 14, 19, and 23, it can be found that the failure of composite laminates with large openings under uniaxial tensile load is a brittle fracture process. The ultimate load decreases with the increase of damage aperture under the same ply condition. The nonlinear relationship between displacement and load appears in the load–displacement curve of the $\left[ { \pm {45}} \right]_{5}$ laminate shown in Fig. 23. This is caused by the nonlinear shear effect of composite laminates, which will be specifically analyzed in the next section.

**Figure 24:**
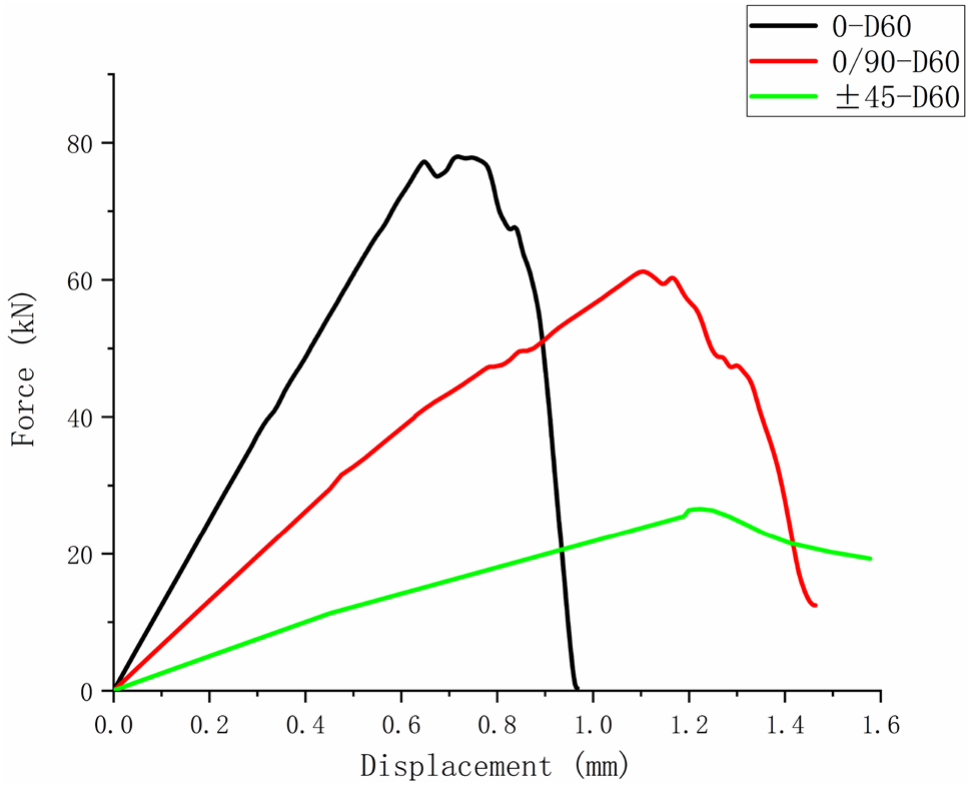
Load displacement curves of three types of laminates with an aperture of 60 mm.

**Figure 25:**
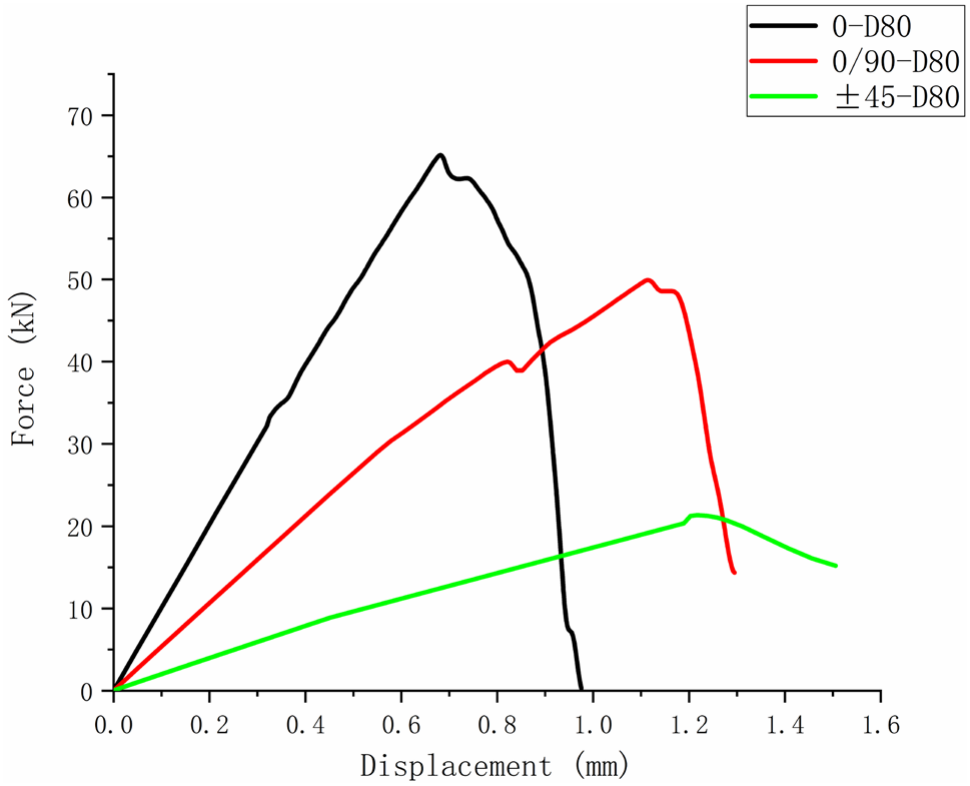
Load displacement curves of three types of laminates with an aperture of 80 mm.

**Figure 26:**
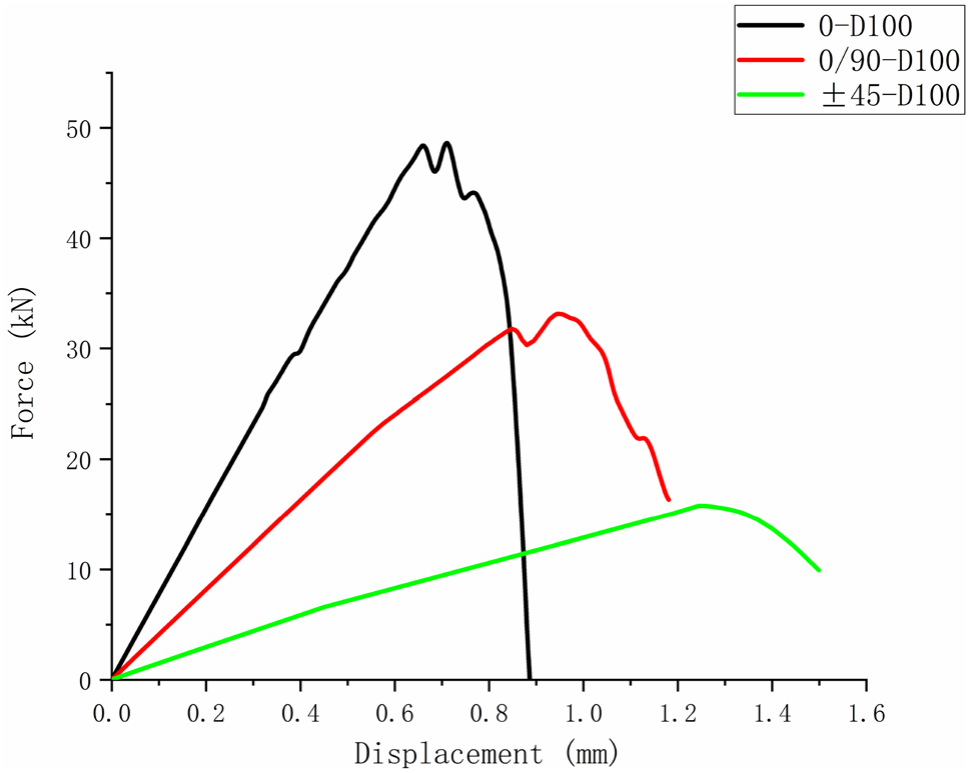
Load displacement curves of three types of laminates with an aperture of 100 mm.

Figures 24, 25, and 26 show the load–displacement curves of three types of laminates with apertures of 60 mm, 80 mm, and 100 mm, respectively. It can be found that under the same type of hole damage, the ultimate load of $\left[ 0 \right]_{10}$ composite laminate is the highest, followed by $\left[ {0/90} \right]_{5}$ composite laminate, and the ultimate load of $\left[ { \pm 45} \right]_{5}$ composite laminate is the lowest.

### Correction considering nonlinear shear

This section will carry out subroutine correction work that takes into account the nonlinear shear effect in order to accurately fit the nonlinear relationship. And the influence of nonlinear shear effect on the failure of different ply laminates was analyzed by comparing the simulation results.

**Figure 27:**
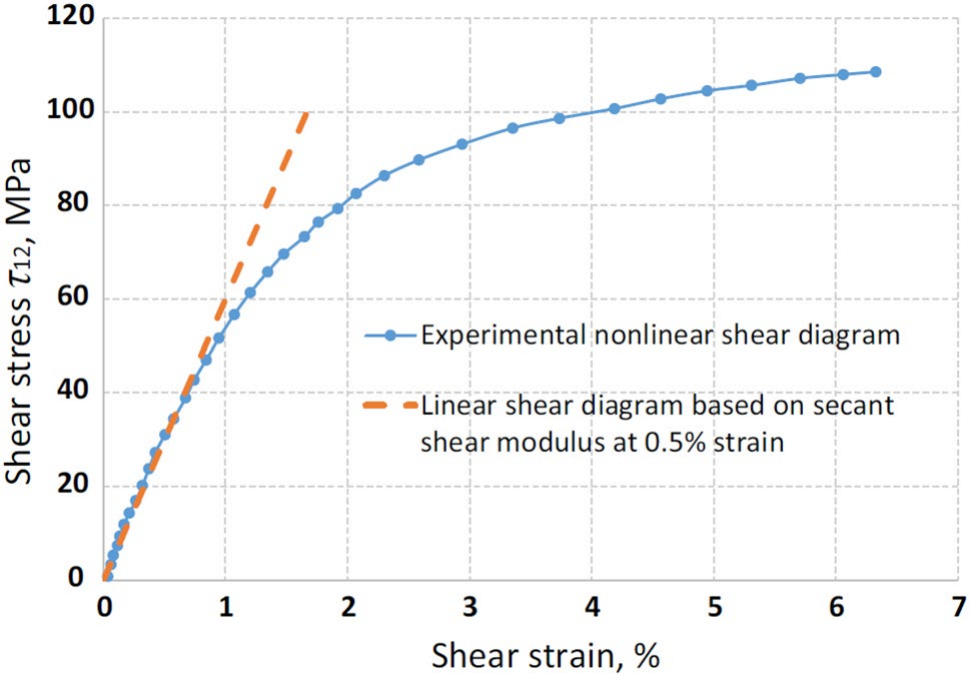
Stress–strain curve of T800/924C laminate in shear test^24^.

A large number of experimental results have been used to demonstrate the nonlinear phenomenon of the stress–strain relationship of composite materials under various types of loads in the study of Puck et al. ^5^. The most common plane shear experiment is the one that best demonstrates the impact of nonlinear effects^23^. Figure 27 shows the nonlinear relationship between shear strain and shear stress obtained from experiments given by Fedulov^24^ et al. It can be clearly seen from the figure that the strain in the nonlinear curve is only relatively close to the linear curve at the initial moment. The limit strain in the nonlinear curve is much larger than the corresponding value of the linear curve as the load increases.

Many scholars have considered nonlinear shear effects in material modeling^25,26^. Liu et al. ^26^ used the Ramberg–Osgood equation^27^ to fit the nonlinear relationship between shear strain and shear stress, with the specific expression as follows:

9 $$\tau = \frac{{G_{0} \gamma }}{{\left[ {1 + \left( {{{G_{0} \gamma } \mathord{\left/ {\vphantom {{G_{0} \gamma } {\tau_{0} }}} \right. \kern-0pt} {\tau_{0} }}} \right)^{n} } \right]^{{{1 \mathord{\left/ {\vphantom {1 n}} \right. \kern-0pt} n}}} }}$$

where $\tau$ and *γ* are shear strain and shear stress, respectively. $G_{0}$ is the initial shear modulus, $\tau_{0}$ is the ultimate shear strength, and *n* is a parameter that defines the shape of the shear nonlinear relationship curve. The expression of shear modulus *G* after considering shear nonlinearity can be obtained from Eq. (9):

10 $$G = \frac{d\tau }{{d\gamma }} = \frac{{G_{0} }}{{\left[ {1 + \left( {{{G_{0} \gamma } \mathord{\left/ {\vphantom {{G_{0} \gamma } {\tau_{0} }}} \right. \kern-0pt} {\tau_{0} }}} \right)^{n} } \right]^{{{1 \mathord{\left/ {\vphantom {1 n}} \right. \kern-0pt} n}}} }}$$

The shear modulus *G* expression shown in Eq. (10) was added to the UMAT subroutine in order to accurately fit the nonlinear effect. Perform simulation analysis on the model in the previous section again to obtain its ultimate load and load–displacement curves.

**Figure 28:**
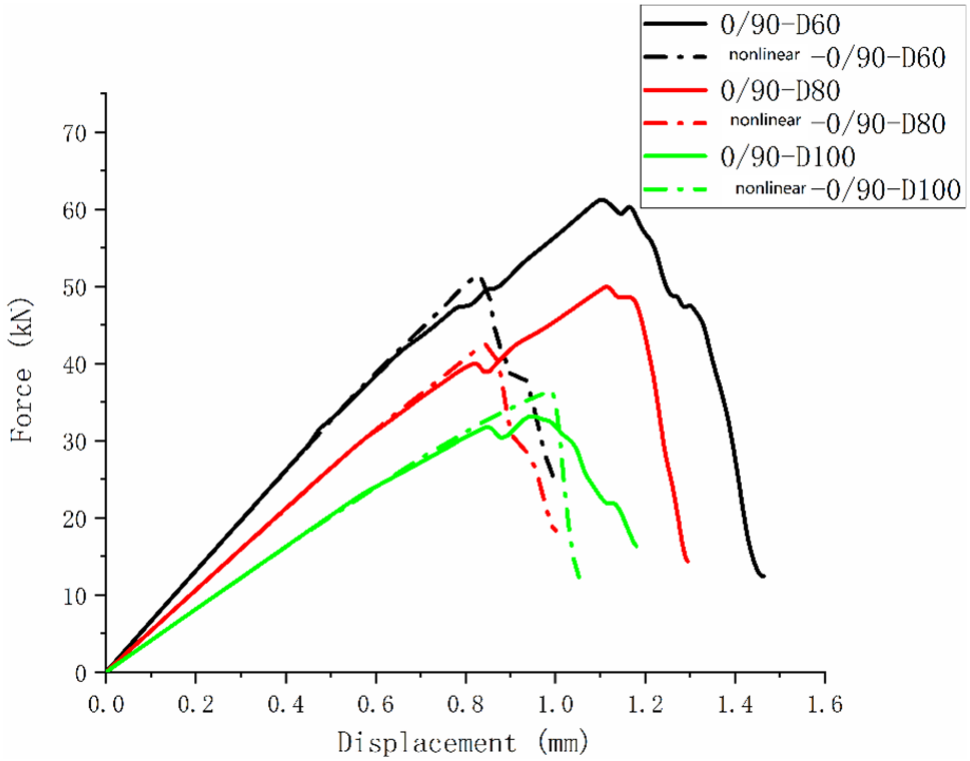
Load–displacement curves of $\left[ {0/90} \right]_{5}$ laminate before and after considering nonlinear shear.

**Figure 29:**
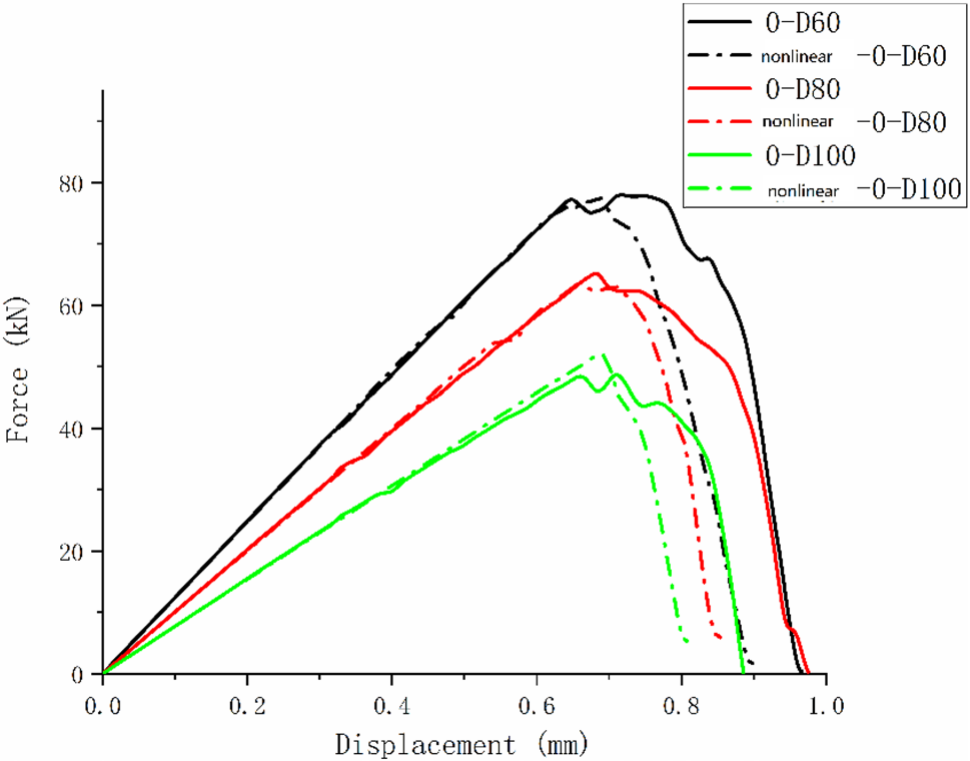
Load–displacement curves of $\left[ 0 \right]_{10}$ laminate before and after considering nonlinear shear.

**Figure 30:**
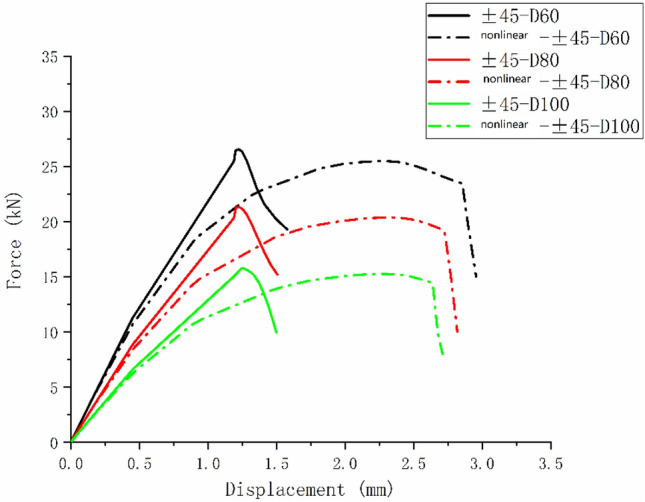
Load–displacement curves of $\left[ { \pm 45} \right]_{5}$ laminate before and after considering nonlinear shear.

Figures 28, 29, and 30 are the load–displacement curves of $\left[ {0/90} \right]_{5}$, $\left[ 0 \right]_{10}$, and $\left[ { \pm 45} \right]_{5}$ composite laminates calculated before and after considering the nonlinear shear effect.

**Figure 31:**
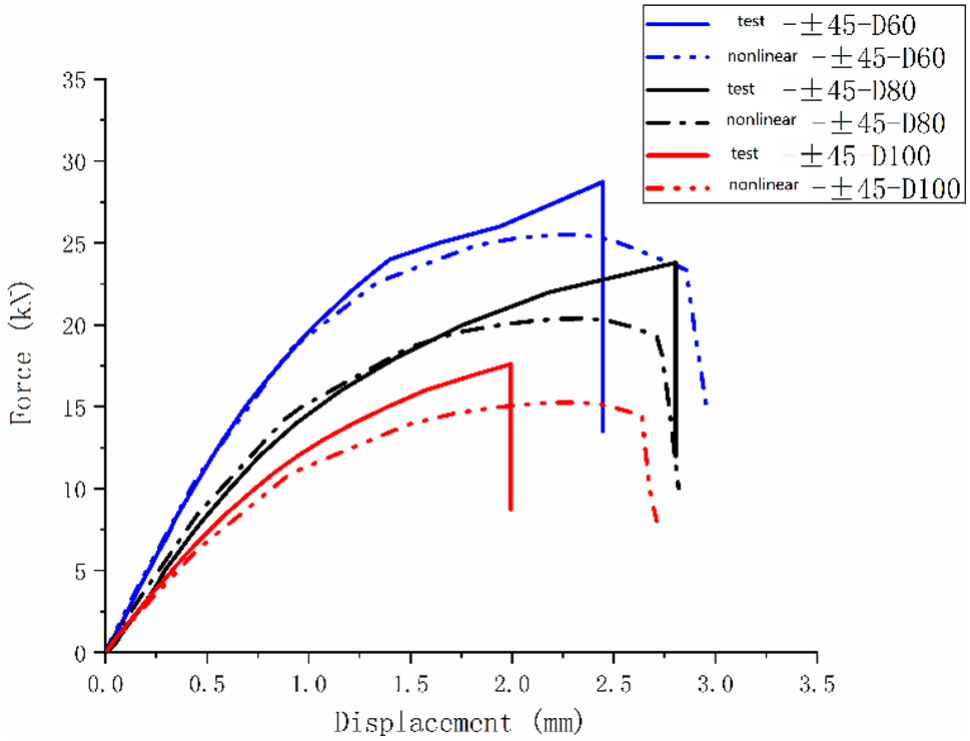
Simulation and experimental load–displacement curves of $\left[ { \pm 45} \right]_{5}$ laminate considering shear nonlinearity.

It can be found from Figs. 28 and 29 that the nonlinear shear effect has little impact on the simulation results of $\left[ {0/90} \right]_{5}$ and $\left[ 0 \right]_{10}$ laminates. It can be found from Fig. 30 that the simulated ultimate load of the $\left[ { \pm 45} \right]_{5}$ laminate decreases after considering the nonlinear shear effect and the failure displacement increases. And the nonlinear relationship between load and displacement becomes more apparent. Figure 31 is a comparison diagram of the simulated and experimental load–displacement curves of $\left[ { \pm 45} \right]_{5}$ laminate calculated considering the nonlinear shear effect^5^.

It can be found from Fig. 31 that the nonlinear relationship in the simulated load–displacement curve is better fitted after considering the shear nonlinear effect. This indicates that the simulation results of $\left[ { \pm 45} \right]_{5}$ laminate considering nonlinear shear effect play a positive role. In the simulation process of this article, due to the fact that the $\left[ { \pm 45} \right]_{5}$ laminates’ failure mode is matrix failure without fiber failure and delamination, the nonlinear performance is particularly evident. Similar results can also be obtained from Refs.^13,16^.

## Conclusion

In this paper, a UMAT subroutine for the progressive damage degradation model of 3D Hashin-Ye failure criterion for composite laminates was compiled and a finite element simulation model was established. The simulation calculation of composite laminate was conducted using UMAT and its damage evolution process was analyzed. The main conclusions obtained by comparing the calculated results with the experiments are as follows.

1. Comparing the simulation results using the ply-discounting degradation model and the continuum damage model with the test results, it was found that both the errors were not significant but the continuum damage degradation model results in smaller error and can better fit the test load displacement curve.
2. The UMAT subroutine using the 3D Hashin-Ye failure criterion and continuum damage degradation model can accurately simulate the failure process of three different plies ($\left[ {0/90} \right]_{5}$, $\left[ 0 \right]_{10}$ and $\left[ { \pm 45} \right]_{5}$) and three different apertures (D = 100 mm, 800 mm, 60 mm) of perforated composite laminates.
3. The failure process of composite laminates with large openings under uniaxial tensile loading was a brittle fracture process and the damage initiation positions were all at the stress concentration points around the holes. The ultimate load of the laminate decreased with the increase of the damage aperture under the same laminate condition. Under the same type of hole damage, the ultimate load of $\left[ 0 \right]_{10}$ composite laminate was the highest, followed by $\left[ {0/90} \right]_{5}$ composite laminate, and the ultimate load of $\left[ { \pm 45} \right]_{5}$ composite laminate was the lowest.
4. A modified shear modulus using the Ramberg Osgood equation was introduced into the constitutive equation considering the effect of nonlinear shear on the failure evolution of composite laminates. Considering the nonlinear shear effect played a positive role in the failure simulation results of $\left[ { \pm 45} \right]_{5}$ diagonal ply laminates. Taking this effect into account can better fit the nonlinear relationship between load and displacement.

## Data Availability

The datasets used and/or analysed during the current study available from the corresponding author on reasonable request.
